# Bone Canopies in Pediatric Renal Osteodystrophy

**DOI:** 10.1371/journal.pone.0152871

**Published:** 2016-04-05

**Authors:** Renata C. Pereira, Thomas L. Andersen, Peter A. Friedman, Navdeep Tumber, Isidro B. Salusky, Katherine Wesseling-Perry

**Affiliations:** 1 Department of Pediatrics, David Geffen School of Medicine at UCLA, Los Angeles, California, United States of America; 2 Department of Clinical Cell Biology (KCB), Vejle Hospital – Lillebaelt Hospital, Institute of Regional Health Science, University of Southern Denmark, Vejle, Denmark; 3 Department of Pharmacology & Chemical Biology, University of Pittsburgh School of Medicine, Pittsburgh, Pennsylvania, United States of America; Université de Lyon - Université Jean Monnet, FRANCE

## Abstract

Pediatric renal osteodystrophy (ROD) is characterized by changes in bone turnover, mineralization, and volume that are brought about by alterations in bone resorption and formation. The resorptive and formative surfaces on the cancellous bone are separated from the marrow cavity by canopies consisting of a layer of flat osteoblastic cells. These canopies have been suggested to play a key role in the recruitment of osteoprogenitors during the process of bone remodeling. This study was performed to address the characteristics of the canopies above bone formation and resorption sites and their association with biochemical and bone histomorphometric parameters in 106 pediatric chronic kidney disease (CKD) patients (stage 2–5) across the spectrum of ROD. Canopies in CKD patients often appeared as thickened multilayered canopies, similar to previous reports in patients with primary hyperparathyroidism. This finding contrasts with the thin appearance reported in healthy individuals with normal kidney function. Furthermore, canopies in pediatric CKD patients showed immunoreactivity to the PTH receptor (PTHR1) as well as to the receptor activator of nuclear factor kappa-B ligand (RANKL). The number of surfaces with visible canopy coverage was associated with plasma parathyroid hormone (PTH) levels, bone formation rate, and the extent of remodeling surfaces. Collectively, these data support the conclusion that canopies respond to the elevated PTH levels in CKD and that they possess the molecular machinery necessary to respond to PTH signaling.

## Introduction

Renal osteodystrophy (ROD) is the term that describes abnormalities in bone turnover, mineralization, and volume [1,2] which are universal in patients with chronic kidney disease (CKD) and which, over time, may lead to increased risk of fractures, bone deformities, and growth failure [3,4]. These abnormalities, diagnosed by bone histomorphometry, are the end result of alterations in bone formation, bone resorption, and matrix mineralization; the degree and type of abnormalities define the different sub-types of ROD.

Cancellous bone remodeling occurs in numerous units scattered over the surface of bone surface. Each unit is comprised of three sequential phases: bone resorption by osteoclasts, a reversal phase characterized by colonization of the eroded surface by pre-osteoblasts (i.e. reversal cells), and bone formation by mature osteoblasts [5–8]. These remodeling events occur on the bone surface and are separated from the marrow cavity by the canopy, consisting of layer of flat osteoblastic cells [9,10]. Canopies are believed to originate from the bone marrow envelope (BME) [7,11], which appear to form a sac around the complete bone marrow [12]. Due to its exceptionally flat appearance and morphological resemblance to bone lining cells, the BME is difficult to identify at the light microscopic level and is only clearly visible by transmission electron microscopy [11]. Only upon transformation into a canopy above remodeling sites, where it is lifted from the bone surface and its cellularity increases [7], does this layer become clearly visible. Importantly, the BME and canopies are believed to reflect a local reservoir of osteoprogenitor cells, which are matured and dispatched to reversal surfaces, ensuring that these surfaces reach the critical cell density needed to transform into a bone-forming surface [7]. In pathological conditions such as multiple myeloma, glucocorticoid-induced osteoporosis and postmenopausal osteoporosis, the absence of this local reservoir of osteoprogenitors has been associated with an insufficient recruitment of osteoprogenitors and an arrest in the reversal phase [9,13–15], preventing transformation of the remodeling unit into its final, bone-formation, phase.

Since CKD is associated with marked changes in bone formation and resorption, and often with a dissociation between the two processes [16], the present study was performed to address the characteristics of canopies in 106 pediatric patients with CKD and to evaluate whether the presence of these structures is associated with biochemical and bone histomorphometric parameters characteristic of ROD.

## Methods

### Patients and biopsies

Bone biopsies and biochemical values were obtained from a de-identified repository of specimens from pediatric patients with CKD stages 2–5. The included biopsies were obtained as part of different research protocols for the assessment of ROD and represent all bone biopsies performed between May 2006 and March 2012 at the University of California at Los Angeles. All biopsies were 5-mm iliac crest bone biopsies, which were dehydrated in alcohol, cleared with xylene, and embedded undecalcified in methyl methacrylate. No patients in the current cohort were treated with immunosuppressive therapy or recombinant human growth hormone for at least 6 months prior to the biopsy and none had undergone a parathyroidectomy within the preceding year. Bone biopsies from individuals with metabolic bone diseases other than ROD were excluded. All subjects had undergone double tetracycline labeling prior to bone biopsies and all samples were obtained from the anterior iliac crest as previously described [17]. The following biochemical variables were determined at bone biopsy: calcium, phosphorus, parathyroid hormone (PTH), albumin, creatinine, alkaline phosphatase, 25(OH)vitamin D and FGF23. Calcium, phosphorus, albumin, creatinine and alkaline phosphatase were measured using an Olympus AU5400 analyzer (Olympus America Incorporated, Center Valley, PA). 25(OH)vitamin D was measured by radioimmunoassay (Heartland Assays, University of Iowa). PTH samples were collected in EDTA plasma and determined by the 1^st^ generation immunometric assay (Immutopics^R^, San Clemente, California, normal range: 10–65 pg/ml). FGF23 was measured in plasma by the 2^nd^ generation C-terminal assay (Immutopics^R^, San Clemente, California). Since biopsies were de-identified, no informed consent was obtained from study participants. The University of California at Los Angeles Institutional Review Board approved the study.

### Bone histomorphometry

Static histomorphometric parameters were evaluated in undecalcified 5 μm sections treated with Toluidine blue; tetracycline labeling was assessed in unstained 10 μm sections. Primary bone histomorphometric parameters were assessed in trabecular bone under 200x magnification using the OsteoMetrics^R^ system (OsteoMetrics, Decatur, GA) by a histomorphometrist (RP). Mineralized bone was defined by dark blue staining areas; pale-blue seams at least 1.5 μm in width were included in measurements of osteoid. Derived indices were calculated according to standard formulae [18]. Since the majority of bone surface in many of the patients with high turnover ROD is actively involved in bone formation or resorption, complicating the assessment of quiescent basic multicellular unit wall thickness, activation frequency was not measured. Patients were classified according to the turnover, mineralization, and volume (TMV) classification system [1,2]. The trabecular bone volume was normal to increased in all patients; thus, bone histologic lesions were classified as 1) low bone turnover (bone formation rate below the normal range, no fibrosis, and osteoid volume/bone volume less than 12%), 2) osteomalacia (bone turnover in the low or normal range in combination with osteoid volume greater than 12%), 3) normal bone turnover (bone formation rate in the normal range with osteoid volume less than 12%), 4) mild secondary hyperparathyroidism (bone formation rate above the normal range and osteoid volume less than 12%), 5) osteitis fibrosa (bone formation rate above the normal range, marrow fibrosis, and osteoid volume less than 12%), or 6) mixed uremic osteodystrophy (bone formation rate above the normal range and osteoid volume greater than 15%) (19). The normal parameters of bone histomorphometry were originally defined from bone biopsies obtained from a control group of 31 pediatric patients with normal kidney function (mean age 12.4 ± 1.5 years; 71% male, 48% Caucasian and 26% Hispanic) who were undergoing elective orthopedic surgery (20). These control values were used as reference ranges only and not included in the analysis.

### Canopy assessment

A trained observer who was blinded with respect to the corresponding biochemical determinations evaluated canopies. Canopies were quantified on a subjective scale of 0 to 4 (**0**: no surfaces with a visible canopy; **0.5**: 1–2 surfaces; **1**: 3–4 surfaces; **2**: 5–6 surfaces; **3**: 7–9 surfaces; **4**: ≥ 10 surfaces).

### Immunohistochemistry

To assess whether canopies have the molecular machinery necessary to transmit and coordinate PTH-mediated signaling, immunoreactivity to PTH receptor-1 (PTHR1) and receptor activator of nuclear factor kappa-B ligand (RANKL) were assessed in all biopsy specimens. The technique for immunohistochemical detection of protein in bone was adapted from a previously reported method [19]. In brief, 5-μm thick sections of bone tissue were de-plasticized in xylene and chloroform, rehydrated in graded alcohol solutions, and partially decalcified in 1% acetic acid. Endogenous peroxidase activity was quenched in 3% hydrogen peroxide/methanol solution. Non-specific binding was blocked in avidin-biotin solution and in 5% normal horse serum with 1% bovine serum albumin. Sections were incubated with affinity purified polyclonal rabbit anti-human PTHR1 [20] (P Friedman laboratory) (dilution 1:250) or polyclonal goat anti-human receptor activator of nuclear factor kappa-B ligand (RANKL) (sc-7628, Santa Cruz Biotechnology, Dallas, TX) (dilution 1:150) primary antibody overnight at 4°C in a humidified chamber. Sections were then incubated with biotinylated anti-goat (Sigma-Aldrich, St. Louis, MO) or anti-rabbit (Sigma-Aldrich, St. Louis, MO) antibody; incubated for 30 minutes with StreptABC Complex/HRP kit (Vector Biolabs, Malvern PA) followed by AEC substract Chromogen (Dako, Carpinteria, CA); and counterstained with Mayer hematoxilin (Sigma-Aldrich). Negative controls were performed on sections from each bone core by omitting the primary antibody. The presence of PTHR1 immunoreactivity in undecalcified bone was quantified (RCP) was quantified on the following subjective scale: **0**: no PTHR1 immunoreactivity; **0.5**: 1–2 surfaces with PTHR1 immunoreactivity; **1**: 3–4 surfaces with PTHR1 immunoreactivity; **2**: 5–6 surfaces with PTHR1 immunoreactivity; **3**: 7–9 surfaces with PTHR1 immunoreactivity; **4**: ≥ 10 surfaces with PTHR1 immunoreactivity. Co-localization for PTHR1 and RANKL was assessed in a subset of 2 samples. After primary antibody incubation for PTHR1 and RANKL, sections were incubated with Alexa Fluor 488 conjugated donkey anti-rabbit and Alexa Fluor 594 conjugated donkey anti-goat antibodies (Abcam, Cambridge, UK), respectively. Sections were then visualized under fluorescence for the presence of PTHR1 immunoreactivity (green), RANKL immunoreactivity (red), and for co-localization of the two proteins (yellow).

### Statistical analysis

Measurements for normally distributed variables are reported as mean ± standard deviation; median values and interquartile range are used to describe non-normally distributed variables. Spearman correlation coefficients were used to assess the relationship between biochemical variables, canopy coverage, and PTHR1 immunoreactivity. Wilcoxon signed rank tests were used to assess differences in biochemical and bone parameters between pre-dialysis CKD and dialysis patients and to assess differences in PTH levels and bone histologic parameters between patients with canopy coverage as compared to those with no apparent canopy coverage. Multiple linear regression was performed to assess the independent contributions of PTH, osteoid surfaces and eroded surfaces to canopy coverage. All statistical analyses were performed using SAS software (SAS Institute Inc., Cary, NC) and all tests were two-sided. A probability of type I error less than 5% was considered statistically significant and ordinary *p* values are reported.

## Results

### Patient demographics and biochemical values

Bone biopsy cores were analyzed from 106 patients with CKD stages 2–5; 18 cores had been obtained from patients with pre-dialysis CKD stages 2–4 and the other 88 were from patients with CKD stage 5 treated with maintenance dialysis. The average age of subjects at the time of biopsy was 14.8 ± 4.2 years (CKD stages 2–4) and 17.4 ± 3.8 years (maintenance dialysis) respectively. Sixty-seven percent of subjects, irrespective of CKD stage, were male. Thirty-seven percent of pre-dialysis CKD subjects were Caucasian and the remainder were Hispanic; dialysis patients were 11% Caucasian, 76% Hispanic, 8% Black, and 5% Asian. Biochemical values obtained at the time of bone biopsy are displayed in Table 1. Serum calcium levels, 25(OH)vitamin D, and alkaline phosphatase activity did not differ between groups; however, serum phosphorus, plasma PTH, and plasma FGF23 concentrations were higher in dialysis patients.

**Table 1:** Biochemical parameters from pre-dialysis CKD and from dialysis patients.

Biochemical variables	CKD stages 2–4 (n = 18)	Dialysis (n = 88)
Calcium (mg/dL)	9.2 ± 0.8	9.0 ± 0.9
Phosphorus (mg/dL)	4.8 ± 1.3	6.5 ±1.9 *
Alkaline phosphatase (IU/L)	212 ± 161	246 ±235
25(OH)vitamin D (ng/mL)	25.1 ±8.1	24.9 ±17.8
1^st^ generation PTH (pg/mL)	68 (48, 137)	508 (310, 930) *
2^nd^ generation C-terminal FGF23 (RU/ml)	228 (101, 462)	1989 (617, 7345) *

Values are displayed as mean ± standard deviation or median (IQ range). The asterisk indicates a difference (p<0.05) between CKD Stages 2–4 and dialysis patients.

### Bone histomorphometry and canopy quantification

**Fig 1:**
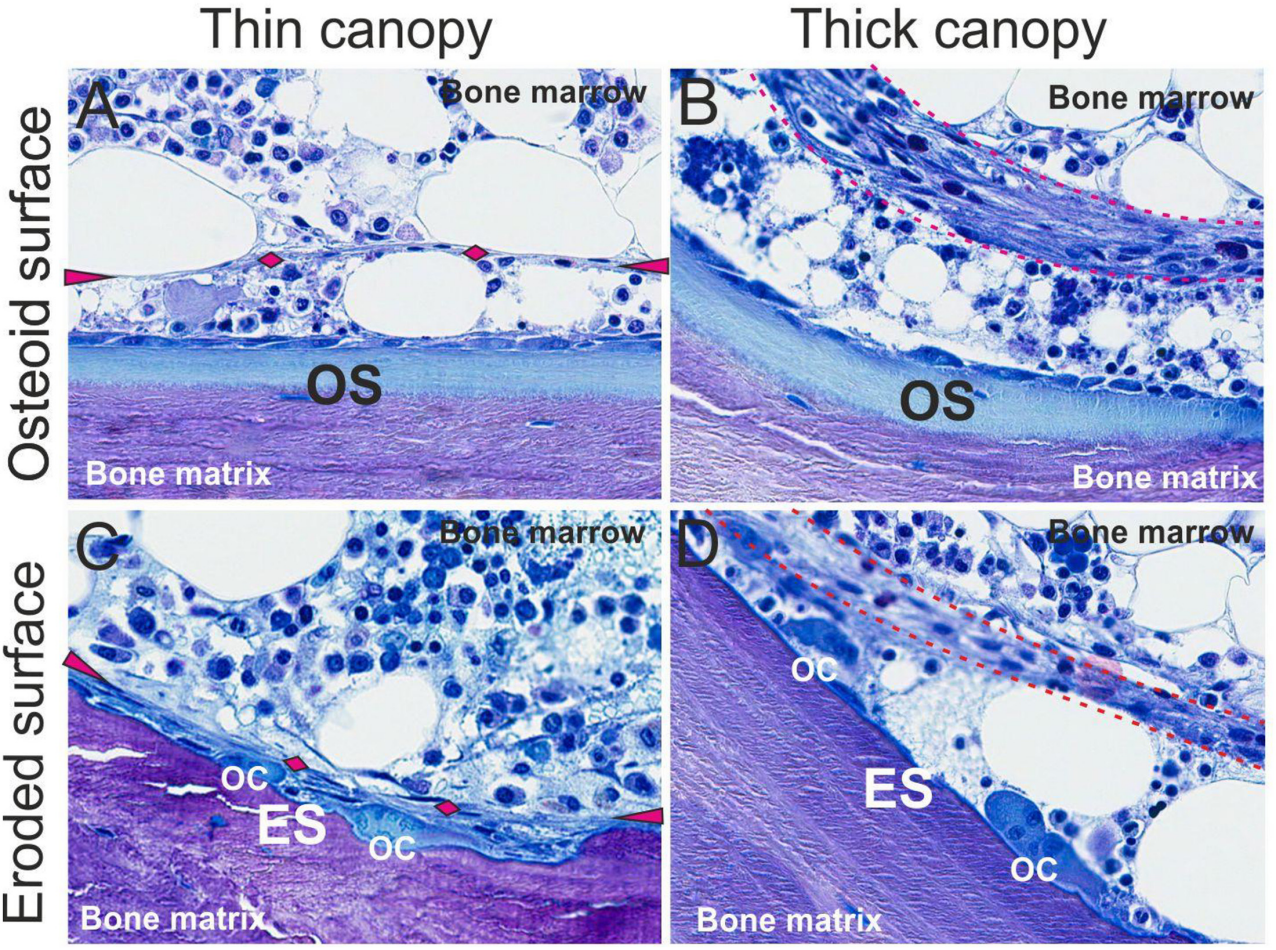
Light microscopic features of canopies in patients treated with maintenance dialysis. Toluidine blue staining of a 5 μm undecalcified section of iliac crest trabecular bone from patients treated with maintenance dialysis demonstrating osteoid surfaces (OS) colonized by osteoblasts (A-B) and eroded surfaces (ES) colonized by osteoclasts (OC) and reversal cells (C-D), which are separated from the bone marrow by either a thin (A, C) or thick (B, D) canopy. The thin canopies are marked with cyan arrowheads and diamonds, while the thick canopies are framed by two cyan dotted lines.

Bone histomorphometric parameters are displayed in Table 2. Pre-dialysis CKD patients had low bone turnover (22%), osteomalacia (11%), normal bone turnover (56%), mild secondary hyperparathyroidism (5%), and mixed uremic osteodystrophy (6%), whereas the distribution of ROD in the dialysis patients was as follows: low bone turnover (20%), normal bone turnover (19%), mild secondary hyperparathyroidism (28%), osteitis fibrosa (11%), and mixed uremic osteodystrophy (20%). As expected, osteoid surfaces (OS/BS), osteoclast surfaces (Oc.S/BS), eroded surfaces (ES/BS), and reversal surfaces (Rv.S/BS) overall were higher in patients treated with maintenance dialysis than in those with pre-dialysis CKD. Canopies were present in bone cores from patients at all stages of CKD. Similar to patients with normal kidney function [10], canopies were observed over osteoid and eroded surfaces in trabecular bone (Fig 1). In contrast to the usually very thin canopies that have been described in patients with normal renal function [10], canopies from cores of CKD patients were often thickened and comprised of multiple cell layers. A greater number of surfaces were observed to be covered by a canopy in iliac crest bone cores from dialysis patients than in cores from their pre-dialysis CKD counterparts (Table 2).

**Table 2:** Bone histomorphometry, PTHR1 expression, and canopy coverage in bone specimens from pre-dialysis CKD and from dialysis patients.

Parameter	CKD stages 2–4 (n = 18)	Dialysis (n = 88)
Histomorphometry		
Bone volume (BV/TV) (%)	29.9 ± 8.5	33.5 ±9.4
Osteoid volume (OV/BV) (%)	5.0 ±9.8	5.1 ±4.7
Osteoid surface (OS/BS) (%)	19.7 ±15.3	31.7 ±16.9 *
Osteoid thickness (O.Th) (μm)	9.9 ±7.2	11.1 ±10.3
Mineral apposition rate (MAR) (d)	0.71 ±0.30	1.04 ±2.06
Mineralizing surface/bone surface (MS/BS) (%)	6.32 ±3.8	10.22 ±8.0
Osteoid maturation time (OMT) (d)	14.5 (8.4, 19.5)	12.6 (9.6, 16.6)
Mineralization lag time (MLT) (d)	24.6 (11.9, 45.2)	35.5 (21.1, 89.2)
Bone formation rate (BFR/BS) (um^3^/ um^2^/yr)	14.7 (5.4, 21.7)	25.6 (8.0, 63.2)
Eroded surface (ES/BS) (%)	4.6 ±3.4	9.6 ±5.6 *
Osteoclast surface (Oc.S/BS) (%)	0.4 ± 0.4	2.0 ±1.9 *
Reversal surface (Rv.S/BS) (%)	4.2 ±3.0	7.5 ±4.7 *
Reversal surface/osteoclast surface (Rv.S/Oc.S) (%)	9.7 (5.5, 21.0)	4.7 (2.5, 7.1) *
Canopy coverage and PTHR1 immunoreactivity		
PTHR1 immunoreactivity (subjective scale: 0–4)	1.0 (0.5, 2.0)	1.0 (0.5, 2.0)
Canopy coverage (subjective scale: 0–4)	0 (0, 0.5)	0.75 (0.5, 2.0) *

Values are displayed as mean ± standard deviation or median (IQ range). The asterisk indicates a difference (p<0.05) between CKD Stages 2–4 and dialysis patients.

### Characterization of PTHR1 and RANKL immunoreactivity in canopies

**Fig 2:**
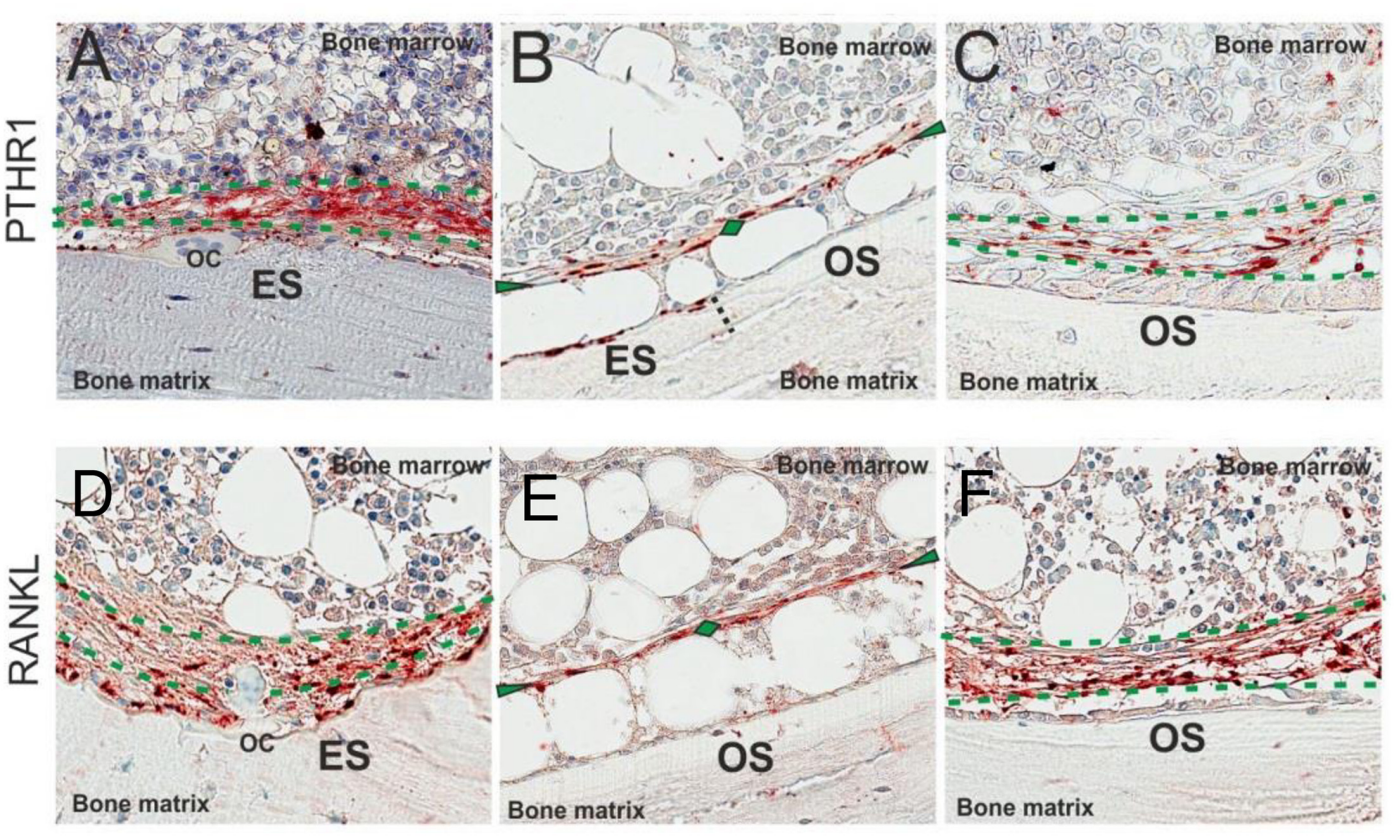
Immunostaining for PTHR1 and RANKL in trabecular bone from a patient treated with maintenance dialysis. A-C: PTHR1 immunoreactivity (red) was observed in both thin and thick canopies above eroded surface (ES) and osteoid surface (OS). On the eroded surface the reversal cells, but not the osteoclasts (OC), demonstrated PTHR1 immunoreactivity (A). No osteoblasts on the osteoid surface showed PTHR1 immunoreactivity (B-C). D-F: RANKL immunoreactivity was also observed in both thin and thick canopies above eroded and osteoid surfaces. Only the reversal cells and not the osteoclasts on the eroded surface demonstrated RANKL immunoreactivity (D). No osteoblasts on the osteoid surface demonstrated RANKL immunoreactivity (E-F). Thin canopies are marked with green arrowheads and diamonds while the thick canopies are framed by two green dotted lines.

**Fig 3:**
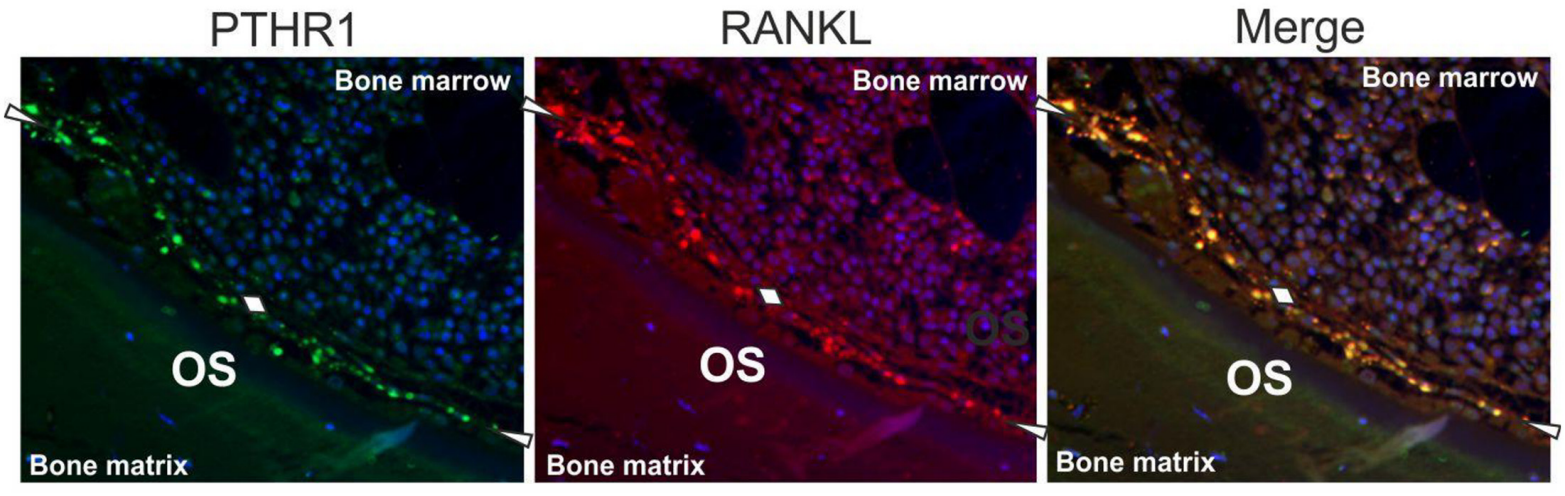
Co-localization of PTHR1 and RANKL in trabecular bone of a dialysis patient. Immunolocalization of PTHR1 (green), RANKL (red), and their co-localization (yellow) in trabecular bone of a patient treated with maintenance dialysis. Thin canopies are marked with white arrowheads and diamonds while thick canopies are framed by two white dotted lines. Note that PTHR1 and RANKL co-localize in canopies.

PTHR1 immunoreactivity was observed in both thin and thick canopies as well as in the reversal cells colonizing the eroded surfaces, while no immunoreactivity was detected in bone forming osteoblasts, or osteoclasts (Fig 2). The number of surfaces with PTHR1 immunoreactivity was similar in both pre-dialysis CKD patients and patients treated with maintenance dialysis (Table 2). Similar to PTHR1, RANKL immunoreactivity was observed in both thin and thick canopies as well as in reversal cells colonizing the eroded surfaces, while nearly no immunoreactivity was observed in osteoblasts or osteoclasts (Fig 2). RANKL immunoreactivity co-localized with PTHR1 immunoreactivity (Fig 3).

### Relationship between canopy coverage, biochemical values, and bone histomorphometry

**Fig 4:**
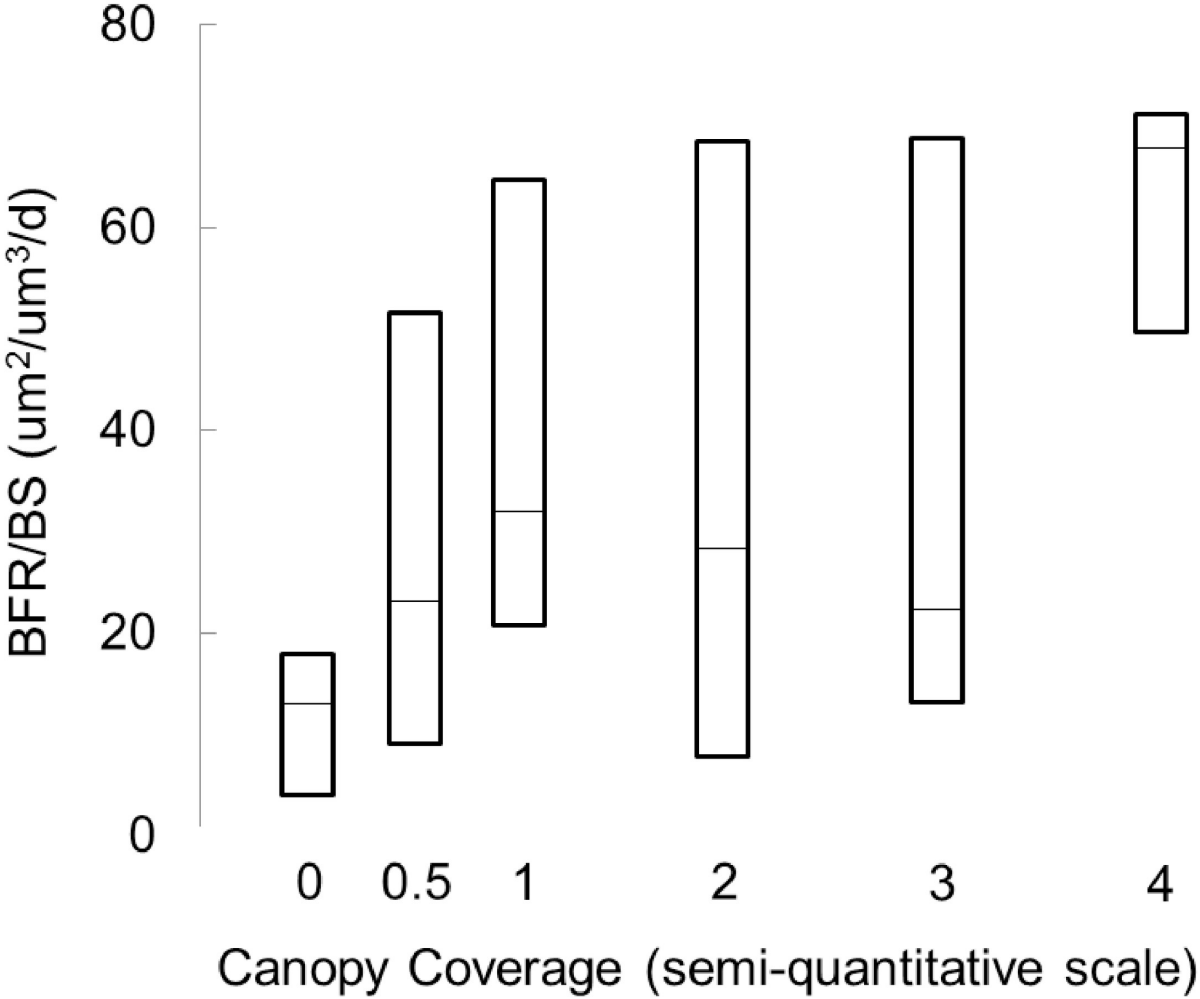
PTH levels as a function of the number of surfaces with canopy coverage in patients with renal osteodystrophy. Lines represent median values; bars represent interquartile ranges.

**Fig 5:**
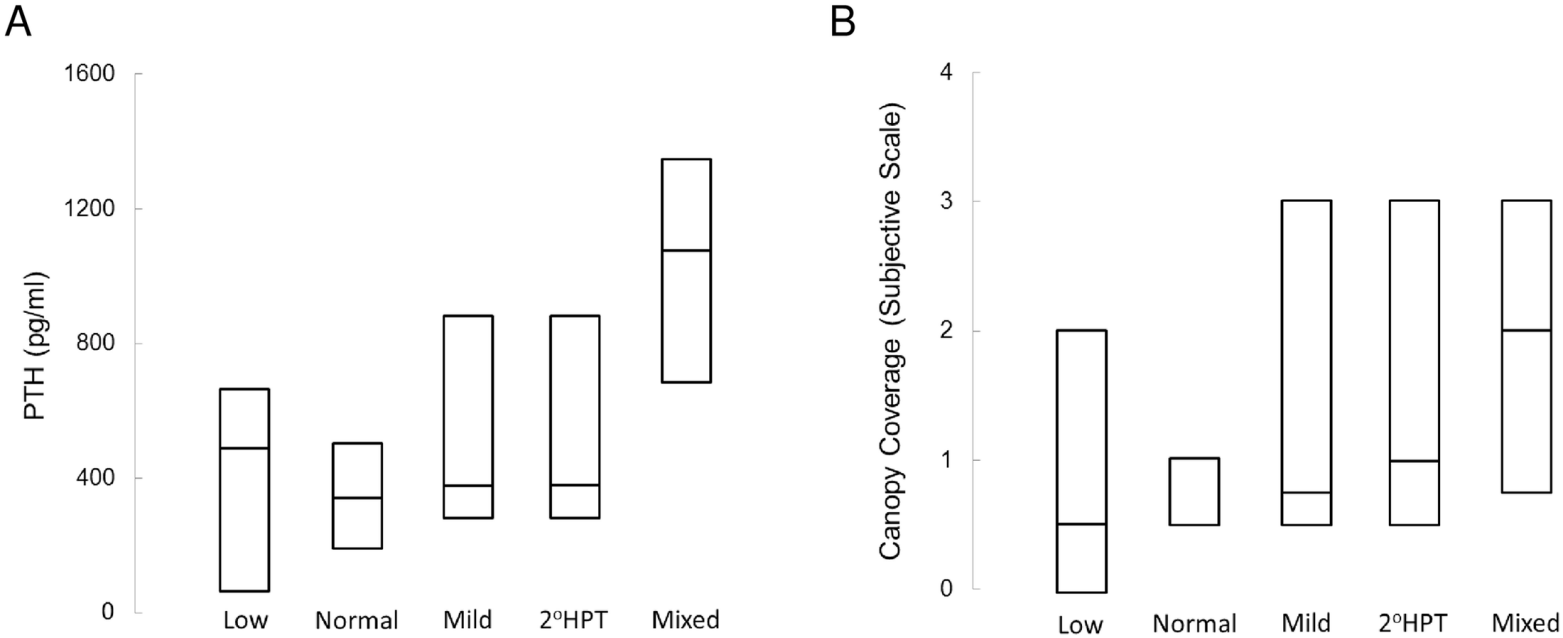
PTH levels (A) and the number of surfaces with canopy coverage (B) as a function of ROD diagnosis in dialysis patients. Lines represent median values; bars represent interquartile ranges. Low: lone bone turnover; normal: normal bone turnover; mild: mildly increased bone turnover; 2°HPT: severe high bone turnover/secondary hyperparathyroidism; mixed: mixed uremic osteodystrophy.

When dialysis and pre-dialysis CKD patients were considered as two separate groups, circulating PTH levels correlated with the number of surfaces with canopy coverage observed in the biopsies (dialysis: r = 0.35, p<0.01; pre-dialysis CKD: r = 0.68, p<0.01). Considering pre-dialysis CKD and dialysis patients together, this relationship was similar (r = 0.54, p<0.01) (Fig 4). As has been previously described [2], there was a good deal of overlap in PTH levels in dialysis patients with different sub-types of ROD (Fig 5a); consistent with their relationship to PTH levels, canopy coverage did not differ in the subtypes of ROD (Fig 5b).

**Fig 6:**
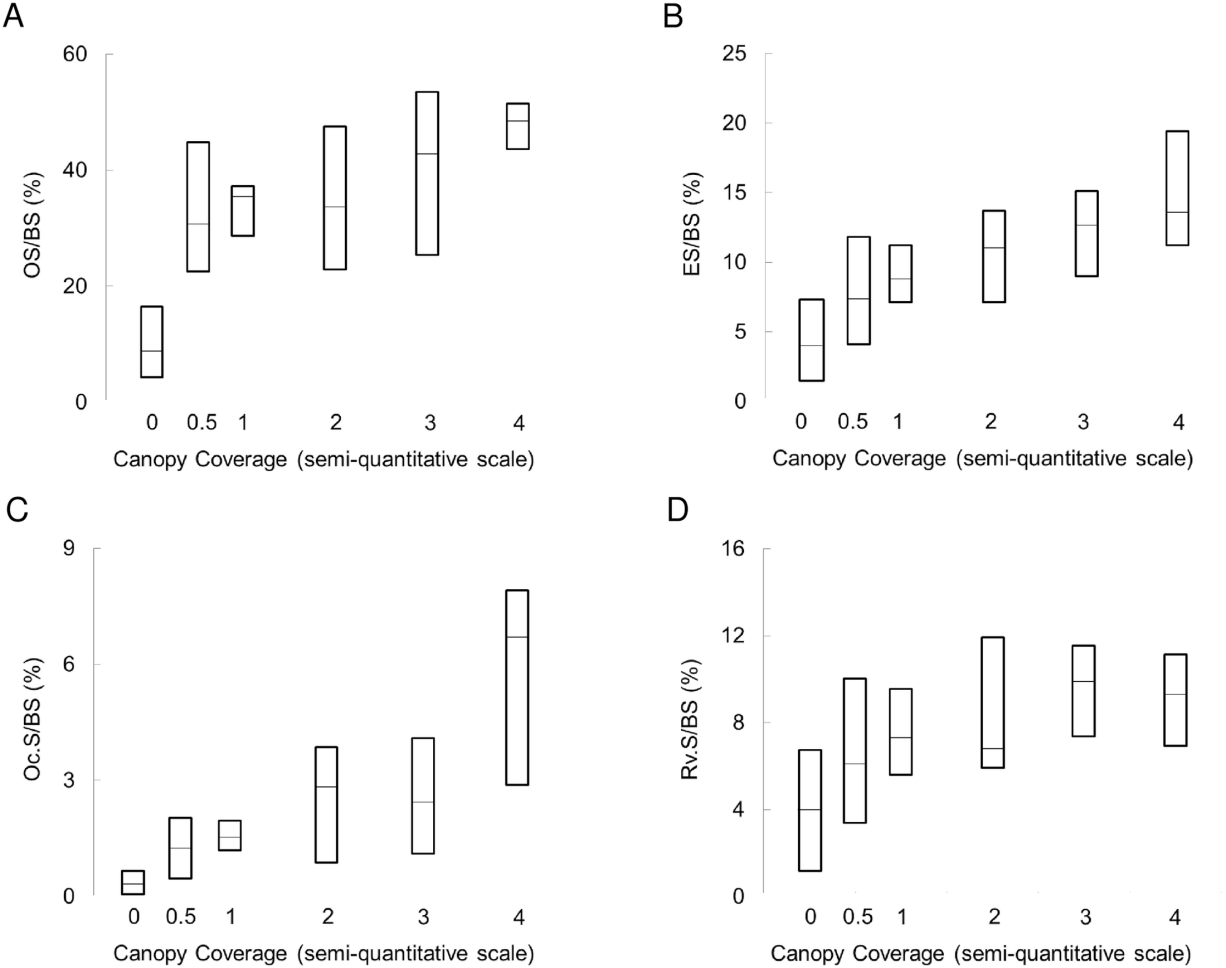
Bone histomorphometric parameters of bone formation and resorption as a function of canopy coverage observed by histology. A) osteoid surfaces/bone surface (OS/BS); B) eroded surface/bone surface (ES/BS). C) osteoclast surface/bone surface (Oc.S/BS); D) reversal surface/bone surface (Rv.S/BS). Lines represent median values; bars represent interquartile ranges.

**Fig 7:**
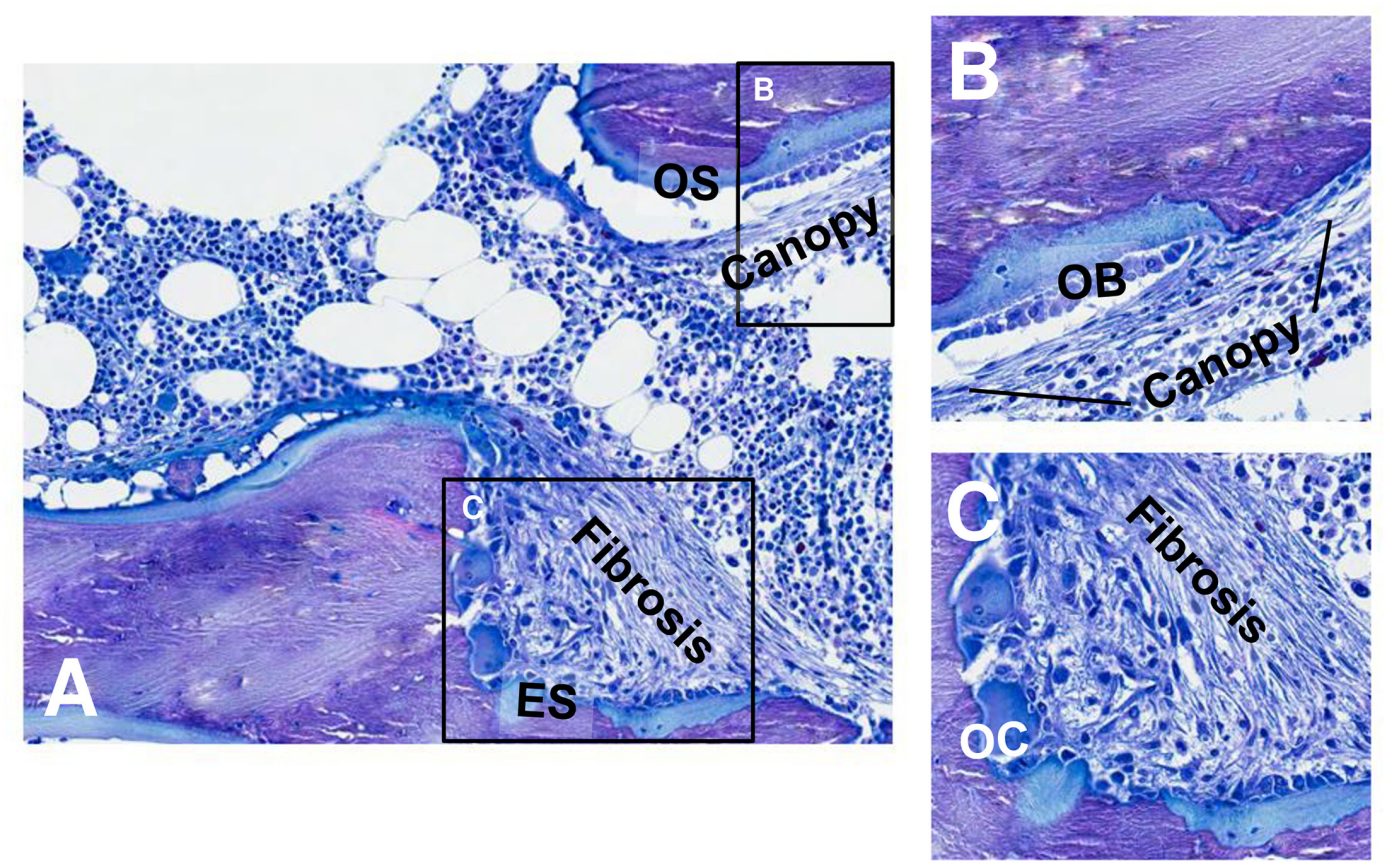
Histologic features of fibrosis and canopies. A) Histologic section demonstrating areas of bone formation (osteoid surface; OS) and resorption (eroded surface; ES). B) Higher magnification of area of bone formation demonstrating organized, layered appearance of canopy overlying bone forming osteoblasts (OB). C) Disorganization characteristic of fibrosis overlying bone-resorbing osteoclasts (OC).

Since the relationship between the number of surfaces with canopy coverage and all other bone histomorphometric parameters were similar in pre-dialysis CKD as compared to dialysis patients, all patients were considered together as one group for subsequent analyses (Table 3). Both the number of surfaces with canopy coverage and with PTHR1 immunoreactivity correlated with bone formation rate (BFR/BS) (r = 0.31, p<0.01 and r = 0.30, p<0.01, respectively). Reflecting the relationship of canopy coverage to overall extent of bone formation and resorption, the number of surfaces with canopy coverage and with PTHR1 immunoreactivity correlated with the extent of osteoid surfaces (OS/BS) (r = 0.58, p<0.01 and r = 0.43, p<0.01) and eroded surfaces (ES/BS) (r = 0.55, p<0.01 and r = 0.31, p<0.01, respectively). Similarly, the number of surfaces with canopy coverage and with PTHR1 immunoreactivity correlated with both the extent of osteoclast surfaces (Oc.S/BS) (r = 0.67, p<0.001 and r = 0.32, p<0.01, respectively) and reversal surfaces (Rv.S/BS) (r = 0.54, p<0.01 and r = 0.44, p<0.01, respectively) (Fig 6). While canopies were observed in patients across the spectrum of renal osteodystrophy, the presence fibrosis is found exclusively in patients with high bone turnover. Histologic feature of fibrosis included a disorganized structure, contrasting with the organized, layered appearance of canopies. Canopies were present over areas of bone resorption and formation, while fibrosis was observed only over eroded surfaces (Fig 7). In the current data set, high bone turnover/secondary hyperparathyroidism with marrow fibrosis was present in 21 patients. In these individuals fibrosis volume correlated with PTH levels (r = 0.79, p<0.001) and with the number of surfaces with canopy coverage (r = 0.63, p<0.01) but not with vitamin D levels (r = −0.20, p = 0.79) or with the interaction between PTH and vitamin D. In multiple linear regression, in which the PTH level, and the extent of osteoid and osteoclast surfaces were considered potential predictors of the number of surfaces with canopy coverage, only the extent of osteoid and osteoclast surfaces remained independent predictors of canopy coverage with an adjusted R^2^ of 51% (Table 4).

**Table 3:** Relationships between biochemical/bone histormorphometric variables and the number of surfaces with canopy coverage or PTHR1 immunoreactivity.

Parameter	Canopy coverage	PTHR1 immunoreactivity
Biochemical Values		
Calcium (mg/dl)	r = −0.11, p = 0.27	r = 0.29, p<0.01
Phosphorus (mg/dl)	r = 0.08, p = 0.47	r = −0.14, p = 0.21
Alkaline phosphatase (IU/l)	r = 0.35, p<0.01	r = 0.52, p<0.01
PTH (pg/ml)	r = 0.54, p<0.01	r = 0.05, p = 0.65
FGF23 (RU/ml)	r = 0.27, p = 0.02	r = −0.03; p = 0.77
Histomorphometry		
Bone volume (BV/TV) (%)	r = 0.15, p = 0.15	r = −0.07, p = 0.47
Osteoid volume (OV/BV) (%)	r = 0.51, p<0.01	r = 0.47, p<0.01
Osteoid surface (OS/BS) (%)	r = 0.58, p<0.01	r = 0.43, p<0.01
Osteoid thickness (O.Th) (μm)	r = 0.39, p<0.01	r = 0.36, p<0.01
Osteoid maturation time (OMT) (d)	r = 0.11, p = 0.32	r = 0.13, p = 0.22
Mineralization lag time (MLT) (d)	r = 0.17, p = 0.12	r = 0.17, p = 0.11
Bone formation rate (BFR/BS) (um^3^/ um^2^/yr)	r = 0.30, p<0.01	r = 0.31, p<0.01
Eroded surface (ES/BS) (%)	r = 0.55, p<0.01	r = 0.31, p<0.01
Osteoclast surface (Oc.S/BS) (%)	r = 0.67, p<0.01	r = 0.32, p<0.01
Reversal surface (Rv.S/BS) (%)	r = 0.54, p<0.01	r = 0.44, p<0.01
Fibrosis volume (Fb.V/BV)	r = 0.63, p<0.01	r = 0.10, p = 0.66

Spearman correlation coefficients and p values are reported.

**Table 4:** Multiple linear regression model considering the contributions of PTH levels, and extent of osteoid and osteoclast surfaces in the prediction of the canopy coverage.

Variable	Parameter Estimate	Standard Error	p value
PTH	0.00021	0.00027	0.43
OS/BS	0.019	0.007	0.01
Oc.S/BS	0.330	0.066	<0.001

Osteoid surface/ bone surface: OS/BS; osteoclast surface/bone surface: Oc.S/BS; Adjusted R^2^ for the model: 0.51.

## Discussion

The current study demonstrates that canopies are present above resorption and formation sites in children with CKD and that these canopies may play a role in the resorption and formation response to increased PTH levels in these young CKD patients.

Bone resorption and formation occurs on cancellous bone surfaces, which are separated from the marrow cavity by a canopy of elongated cells, as recently in reported in healthy adults, in adult patients with multiple myeloma [9], and in adult patients with glucocorticoid-induced [14,15] and postmenopausal osteoporosis [13]. In contrast to adults with normal kidney function, canopies in children with CKD appeared not only as thin structures with few cell layers, but were often very thick and composed of multiple cell layers. Interestingly, similar multi-layered canopies have been observed in adult patients with primary hyperparathyroidism [10], who, like many young CKD patients also have high circulating PTH levels. In this context, it is interesting to note that the canopies in CKD patients have immunoreactivity for PTHR1 and that the number of surfaces with canopy coverage correlates with the circulating PTH levels, as do the extent of both eroded and formative surfaces. PTH has indeed been reported both to have a catabolic and an anabolic effect on the bone, depending on whether the exposure is continuous or intermittent [21–23]. Our observations imply that canopies are likewise affected by PTH levels, a suggestion that is supported by findings in adult patients with primary hyperparathyroidism, in whom parathyroidectomy resulted in a 50% decrease in the proportion cancellous bone surface with canopy coverage [10]. Since canopies have been suggested to be local reservoirs of osteoprogenitors that are recruited to the surface prior and during the bone formation [7,11], the finding of PTH receptor immunoreactivity within canopy cells suggests that they may play a critical role in mediating the effect of PTH on the bone formation. The direct relationship between serum PTH levels and the number of surfaces with PTHR1 immunoreactivity further suggests that PTH may stimulate expression of its own receptor. Future studies, including patients treated with intermittent PTH and those treated with continuous PTH infusion, are warranted to clarify further the role of the canopies in mediating this signal cascade.

The current study also highlights the importance of evaluation bone biopsies for the presence of canopies when evaluating marrow fibrosis in patients with CKD. Indeed, canopies, and particularly multi-layered canopies, may be mistaken for marrow fibrosis in these individuals. It is important to note that while fibrosis is found exclusively in patients with high bone turnover, canopies can be identified in CKD patients with low, normal, and high bone turnover. The histologic features of fibrosis include a disorganized structure, which contrasts with the organized, layered appearance of canopies. In patients with severe secondary hyperparathyroidism, it can be difficult to distinguish where canopies leave off and fibrosis takes over; however, it should be noted that canopies, in contrast to fibroblasts, are noted overlying osteoblasts and demonstrate immunostaining for PTHR1 and RANKL.

A potential role for canopies in mediating the catabolic effects of PTH remains an additional open question. In the current study, combined immunofluorescence staining for PTHR1 and RANKL was performed on a subset of the biopsies. This staining revealed that some canopies displayed immunoreactivity for both PTHR1 and RANKL, the main factor promoting both osteoclastogenesis and osteoclast function [24]. This is consistent with the concept that RANKL is expressed by osteoblast-lineage cells and the idea that osteoblast-lineage cells mediate osteoclastic responsiveness to PTH [24]. This also suggests that canopies have the ability support the differentiation of pre-osteoclasts into mature osteoclasts and to promote the activity of the mature osteoclasts. Future studies addressing the mRNA expression of RANKL will be needed to validate the presence of RANKL in canopies.

In conclusion, canopies are present in pediatric CKD patients. The canopy structures in these patients often appear thickened and consist of multiple cell-layers; this finding differs from their very thin appearance in patients with normal kidney function. The presence of canopies is associated with the circulating PTH levels in pediatric CKD patients and canopies express the molecular machinery required to respond directly to PTH. An open question remains as to whether the canopies play a critical role in the anabolic and catabolic effect of PTH in the pathogenesis of ROD.
