# Correction: Bone Canopies in Pediatric Renal Osteodystrophy

**DOI:** 10.1371/journal.pone.0155663

**Published:** 2016-05-11

**Authors:** Renata C. Pereira, Thomas L. Andersen, Peter A. Friedman, Navdeep Tumber, Isidro B. Salusky, Katherine Wesseling-Perry

Fig 4 appears incorrectly in the published article. Please see the correct Fig 4 and its caption below.

**Fig 4:**
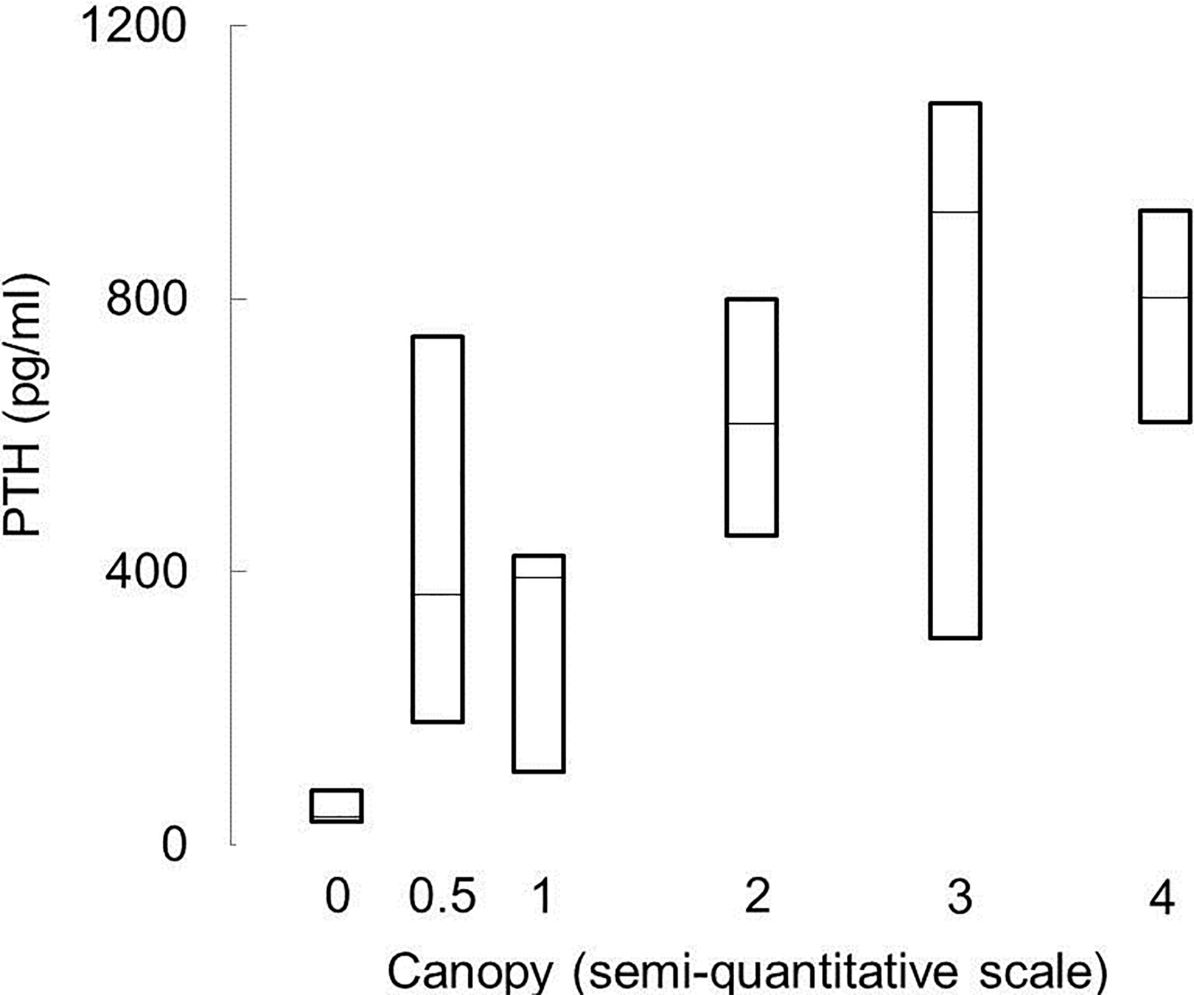
PTH levels as a function of the number of surfaces with canopy coverage in patients with renal osteodystrophy. Lines represent median values; bars represent interquartile ranges.
